# Enabling Large-Scale Biomedical Analysis in the Cloud

**DOI:** 10.1155/2013/185679

**Published:** 2013-10-31

**Authors:** Ying-Chih Lin, Chin-Sheng Yu, Yen-Jen Lin

**Affiliations:** ^1^Master's Program in Biomedical Informatics and Biomedical Engineering, Feng Chia University, No. 100 Wenhwa Road, Seatwen, Taichung 40724, Taiwan; ^2^Department of Applied Mathematics, Feng Chia University, No. 100 Wenhwa Road, Seatwen, Taichung 40724, Taiwan; ^3^Department of Information Engineering and Computer Science, Feng Chia University, No. 100 Wenhwa Road, Seatwen, Taichung 40724, Taiwan; ^4^Department of Computer Science, National Tsing Hua University, No. 101, Section 2, Kuang-Fu Road, Hsinchu 30013, Taiwan

## Abstract

Recent progress in high-throughput instrumentations has led to an astonishing growth in both volume and complexity of biomedical data collected from various sources. The planet-size data brings serious challenges to the storage and computing technologies. Cloud computing is an alternative to crack the nut because it gives concurrent consideration to enable storage and high-performance computing on large-scale data. This work briefly introduces the data intensive computing system and summarizes existing cloud-based resources in bioinformatics. These developments and applications would facilitate biomedical research to make the vast amount of diversification data meaningful and usable.

## 1. Introduction

In more and more cases, the ability to gain experimental data has far surpassed the capability in doing further analyses. DNA sequencing presents a particularly good example of this trend. By current next-generation sequencing (NGS) technologies, an individual laboratory can generate terabase-scales of DNA and RNA sequencing data within a day at a reasonable cost [1–3]. However, the computing technologies required to maintain, process, and integrate the massive datasets are beyond the reach of small laboratories and introduce serious challenges even for larger institutes. Success at all fields will heavily rely on the ability to explain these large-scale and great diversification datasets, which drives us to adopt advances in computing methods.

The coming age of sharp data growth and increasing data diversification is a major challenge for biomedical research in the postgenome era. Cloud computing is an alternative to crack the nut because it gives concurrent consideration to enable storage and massive computing on large-scale data [4–6]. More than this cloud platform can considerably save costs in server hardware, administration, and maintenance by the virtualization technology, which allows systems to act like real computers with flexible specification of the number of processors, memory, and disk size, operating system, and so on. With flexible cloud architectures that can harness petabyte scales of data, Internet-based companies, such as Google and Amazon, offer on-demand services to tens of thousands of users simultaneously. In addition, cloud storages allow large-scale and potentially shared datasets to be stored on the same infrastructure where further analyses can be run [7]. A good example is the data from the 1000 Genomes Project, which has grown to 200 terabytes of genomic data including DNA sequenced from more than 1,700 individuals, and it is now available on the Amazon cloud [8]. Developing translational biomedical applications with cloud technologies will enable significant breakthroughs in the diagnosis, prognosis, and high-quality healthcare. This study introduces the data-intensive computing system and summarizes existing cloud-based resources in bioinformatics. These developments and applications would facilitate biomedical research to make the massive datasets meaningful and usable.

This paper is organized as follows.  Section 2 introduces the state of the art in the cloud developments of translational biomedical science. Subsequently, we review the framework and platforms for massive computing in the cloud in Section 3. Finally, Section 4 draws our conclusion.

## 2. Translational Biomedical Science in the Cloud

Over the last decades, biomedical informatics has contributed a vast amount of data. In the genomic side, the data deluge comes from genotyping, gene expression, NGS data, and so on. The sequence read archive (SRA) provides the scientific community with an archival destination for raw sequence data, whose volume has reached 1.6 petabytes in 2013 [9]. A key goal of 1000 Genomes Project is to investigate the genetic contribution to human disease by characterizing the geographic and functional spectrum of genetic variation on a great deal of sequencing data [10]. More genome-wide association studies (GWAS) continue to identify common genetic factors that influence health or cause disease [11–13]. On the other hand, the diagnosis side constantly generates data from pharmacy prescription data, electronic medical and insurance records, healthcare information, and so forth. Electronic health record (EHR) is a digital data for the traditional document-based patient chart and has been essential to manage the wealth of existing clinical information. US health care data alone reached 150 exabytes (=10^9^ gigabytes) in 2011, while at this rate its volume would be zettabyte (=10^12^ gigabytes) scale soon [14]. In many respects, the two sides of biomedical data growth have yet to converge; however, the biomedical infrastructure for big data analysis lags behind the applications. The healthcare system has no capacity yet to distill the implicit meaning of the planet-size data for timely medical decision making. Despite the strong challenge of big data, there are considerable works in the bioinformatics community to develop feasible solutions. In what follows, existing cloud-based resources and GPU computing are summarized to the two types of biomedical data.

### 2.1. Genomic-Driven Data

Today new technologies in genomics/proteomics generate biomedical data with an explosive rate. With data volume getting larger more quickly than traditional storage and computation can afford, it is the time for biomedical studies to migrate these challenges to the cloud. Cloud computing offers new computational paradigms to not only deal with data and analyses at scale but also reduce the building and operation costs. By cloud technologies, numerous works have reported successful applications in bioinformatics (Table 1). These recent developments and applications would facilitate biomedical studies to harness the planet-size data.

Cloud-based tools in Table 1 combine distributed computing and large-scale storage to come with an effective solution in terms of data transfer, storage, computation, and analysis of big biomedical data. By deploying applications with these tools, small laboratories could maintain and process the large-scale datasets within affordable costs, which is increasingly thorny even for large institutes. For example, BioVLab infrastructure [28, 36] built on the cloud is developed for genome analysis by utilizing the *virtual collaborative lab*, a suite of tools that allow scientists to orchestrate a sequence of data analysis tasks using remote computing resources and data storage facilities on demand from local devices. Furthermore, the Crossbow [21] genotyping program applies the MapReduce workflow on Hadoop to launch many copies of the short-read aligner Bowtie [20] in parallel. Once the aligned reads are generated, Hadoop automatically starts the MapReduce workflow of consensus calling to sort and aggregate the alignments. In the benchmark set on the Amazon EC2 cloud, Crossbow genotyped a human sample comprising 2.7 billion reads in less than 3 hours using a 320-CPU cluster for a total cost of $85 [21].

### 2.2. Diagnosis-Driven Data

More and more requirements to the healthcare quality raise difficulties in processing both the heavy and heterogeneous biomedical data. For example, the high-resolution and dynamic data of medicinal images imply that the data transfer and image analysis are extremely time-consuming. Several works leverage the cloud approach to tackle the difficulties. MapReduce, the parallel computing framework in cloud, has been used to develop an ultrafast and scalable image reconstruction method for 4D cone-beam CT [37]. A solution to power the cloud infrastructure for digital imaging communication in medicine (DICOM) is introduced as a robust cloud-based service [38]. Whereas cloud-based medical image exchange is increasingly prevalent in medicine, its security and privacy issues to the data storage and communication need to be improved [39, 40].

An alternative to attack compute-intensive problems relies on the graphics processing unit (GPU), where there are two dominant APIs for GPU computing: CUDA and OpenCL [41]. GPU architectures feature several multiprocessors with each number of stream processors. The kernel is a function on GPU, while it splits works into blocks and threads. Blocks are assigned to run on multiprocessors, each of which is composed of a user-defined number of threads. The number of threads in a block can be different to the number of stream processors inside a multiprocessor because they run in groups of constant threads called warps. Stream processors are similar to CPU cores, but they share a single fetch-decode unit within the same multiprocessor, which forces threads to execute in lockstep. The mechanism likes the traditional single instruction multiple data (SIMD) instruction; however, any thread can diverge from the common execution path so as to increase the flexibility. Two review papers present the works on GPU accelerated medical image processing and cover algorithms that are specific to individual modalities [42, 43]. Intel quite recently unveiled its new Xeon Phi coprocessor as their many integrated core (MIC) product, while the China Tianhe-2 with the coprocessor inside was announced by TOP500 as the world's fastest supercomputer in 2013 [44]. The new coprocessor has a dramatic impact on the high-performance computing field and will drive more bioinformatics applications [45].

As to the clinical informatics, a major challenge is to integrate a wide range of heterogeneous data into a single and space-saving database for further queries and analyses. EHR could be an ideal solution because it is the patient-centered record by integrating and managing personal medical information from various sources. EHRs are built to share information with other healthcare providers and organizations, while the cloud technologies can facilitate EHR integration and sharing. Developing EHR services on the cloud can not only reduce the building and operation costs but also support the interoperability and flexibility [46]. There are a great number of works that contributed different cloud-supported frameworks to improve EHR services. For instance, an e-health cloud system is defined to be capable of adapting itself to different diseases and growing numbers of patients, that is, improving the scalability [47]. Khansa et al. proposed an intelligent cloud-based EHR system and claimed that it has the potential to reduce medical errors and improve patients' quality of life [48]. A recent work introduces the state of cloud computing in healthcare [49]. Moreover, there are a number of security issues/concerns associated with cloud computing, which is one of the major obstacles for the commercial considerations. As the emerging cloud technology to the healthcare system, more recent studies investigate the security and privacy issues [50–53].

## 3. Massive Computing in the Cloud

Cloud computing started with the promise of inexhaustible resources so that the data-intensive computing can be easily deployed. The three service models of cloud computing, that is, Infrastructure-as-a-Service (IaaS), Platform-as-a-Service (PaaS), and Software-as-a-Service (SaaS), drive more complex and sophisticated markets. What makes cloud computing different from traditional IT technologies are mainly service delivery and consumer utilization models. Cloud platform is rapidly growing as a new paradigm for provisioning both storage and computing as a utility [54]. Based on the platforms, the IT capability is raised so that services can be easily deployed in a pay-as-you-go model. Subsequently, lots of resources could be acquired with a relatively low cost to test novel ideas or conduct extensive simulations. One could access more computing resources in lab to carry out his innovation based on a self-service and self-managed environment. Also, the feature of scalability for cloud platforms allows a lab-scale tool to be extended to a cloud application or a data-intensive scalable computing (DISC) system with fewer efforts [55, 56].

### 3.1. MapReduce Framework

One cannot mention DISC without mentioning MapReduce, while even many works regard MapReduce as the de facto standard for DISC [55, 57]. In 2004, Google announced a distributed computing framework, MapReduce, as the key technology for processing large datasets on a cluster made by upwards of one thousand commodity machines [58]. The MapReduce framework facilitates the management and development of massively parallel computing applications. A MapReduce program consists of two user-specified functions: map and reduce. The map function processes a <key, value> pair to generate a set of intermediate pairs, whereas the reduce function merges all intermediate results associated with the same key. In the beginning, the programming framework is used to assist Google in speedy searches, and nowadays more than 10,000 distant programs have been conducted at Google for the large-scale data analysis [57]. Once applications are modeled to the MapReduce manner, they all enjoy the scalability and fault-tolerance inherent in its execution platform supported by Google File System (GFS), whereas the successful implementation of the MapReduce model, the open-source platform Hadoop, along with the MapReduce framework, has been extensively used outside of Google by academia and industry [59]. Moreover, Ekanayake et al. compared the performances of Hadoop MapReduce, Microsoft Dryad-LINQ, and MPI implementation on two bioinformatics applications and suggested that the flexibility of MapReduce will become the preferred approach [60]. Recently, more and more MapReduce applications are proposed for bioinformatics studies [16–18, 33, 37, 61].

### 3.2. Cloud Platform

PaaS provides a substantial boost with the manageable cost, and there have been a number of solutions, such as Google App Engine (GAE), Amazon Elastic Compute Cloud (EC2), and Windows Azure. GAE offers a robust and extensible runtime environment for developing and hosting web-based applications in Google-managed infrastructure, rather than providing direct access to a customized virtual machine. Malawski et al. investigated how to use GAE service for free of charge execution of compute-intensive problems [62], while Prodan et al. compared GAE and Amazon EC2 in performance and resource consumption by four basic algorithms [63]. EC2 is a cloud service whereby one can rent virtual machines from Amazon data center and deploy scalable applications on them. Several works are conducted to evaluate EC2 performance [64]. Wall et al. concluded that the effort to transform existing comparative genomics algorithms from local infrastructures to cloud is not trivial, but the cloud environment is an economical alternative in the speed and flexibility considerations [65]. Further, two works explore the biomedical cloud built on Amazon service with several case studies [66, 67].

Windows Azure platform provides a series of services for developing and deploying Windows-based applications on the cloud, and it makes use of Microsoft infrastructure to host services and scale them seamlessly [68–70]. Moreover, Aneka provides a flexible model for developing distributed applications, which can be integrated with external cloud platforms further. Aneka presents the possibility to avoid vendor lockin through a virtual infrastructure, a private datacentre, or a server, so that one could freely scale to cloud platforms when required. Its deadline-driven provisioning mechanism also supports QoS-aware execution of scientific applications in hybrid clouds [71]. It is handy to leverage famous PaaS platforms for compute-intensive applications; however, commercial cloud services charge for CPU time, storage space, bandwidth usage, and advanced functions. Apart from the service charge, the commercial cloud platform is still difficult for data-intensive applications. The critical factor is that current network infrastructure is too slow to enable terabytes of data to be routinely transferred. A feasible solution for transferring planet-size data is to copy the data into a big storage drive and then send the drive to the destination. In addition, the private cloud solution helps developers to construct cloud platforms for local use [72].

## 4. Conclusions

Recent technologies on next-generation sequencing and high-throughput experiments cause an exponential growth of biomedical data, and subsequently serious challenges arise in processing data volume and complexity. Numerous works have reported successful bioinformatics applications to harness the big data. Developing cloud-based biomedical applications can integrate the vast amount of diversification data in one place and analyze them on a continuous basis. This would make a significant breakthrough to launch a high-quality healthcare. This work briefly introduces the data-intensive computing systems and summarizes existing cloud-based resources in bioinformatics. These developments and applications would facilitate biomedical applications to make the planet-size data meaningful and usable.

## Figures and Tables

**Table 1:** Cloud-based bioinformatics tools.

Program	Description	URL	Reference
Sequence alignment			
Cloud-Coffee	Multiple sequence alignment	http://www.tcoffee.org/	[15]
USM	MapReduce solution to sequence comparison	http://usm.github.io/	[16]

Sequence mapping and assembly			
CloudBurst	Reference-based read mapping	http://cloudburst-bio.sourceforge.net/	[17]
CloudAligner	Short read mapping	http://cloudaligner.sourceforge.net/	[18]
SEAL	Short read mapping and duplicate removal	http://biodoop-seal.sourceforge.net/	[19]
Crossbow	Combine sequence aligner Bowtie and the SNP caller SOAPsnp [20]	http://bowtie-bio.sourceforge.net/crossbow/	[21]
Contrail	*De novo* assembly	http://contrail-bio.sourceforge.net/	[22]
Eoulsan	Sequencing data analysis	http://transcriptome.ens.fr/eoulsan/	[23]
Quake	Quality-aware detection and correction of sequencing errors	http://www.cbcb.umd.edu/software/quake/	[24]

Gene expression			
Myrna	Differential expression analysis for RNA-seq	http://bowtie-bio.sourceforge.net/myrna/	[25]
FX	RNA-seq analysis tool	http://fx.gmi.ac.kr/	[26]
ArrayExpressHTS	RNA-seq process and quality assessment	http://www.ebi.ac.uk/services	[27]

Comprehensive application			
BioVLab	A virtual collaborative lab for biomedical applications	https://sites.google.com/site/biovlab/	[28]
Hadoop-BAM	Directly manipulate NGS data	http://sourceforge.net/projects/hadoop-bam/	[29]
SeqWare	A scalable NoSQL database for NGS data	http://seqware.sourceforge.net	[30]
PeakRanger	Peak caller for ChIP-seq data	http://ranger.sourceforge.net/	[31]
YunBe	Gene set analysis for biomarker identification	http://tinyurl.com/yunbedownload/	[32]
GATK	Genome analysis toolkit	http://www.broadinstitute.org/gatk/	[33]
Cloud BioLinux	A virtual machine with over 135 bioinformatics packages	http://cloudbiolinux.org/	[34]
CloVR	A virtual machine for automated sequence analysis	http://clovr.org/	[35]
